# Overwintering Physiology and Cold Tolerance of the Sunn Pest, *Eurygaster integriceps*, an Emphasis on the Role of Cryoprotectants

**DOI:** 10.3389/fphys.2020.00321

**Published:** 2020-04-30

**Authors:** Hamzeh Hasanvand, Hamzeh Izadi, Mozhgan Mohammadzadeh

**Affiliations:** Department of Plant Protection, Faculty of Agriculture, Vali-e-Asr University of Rafsanjan, Rafsanjan, Iran

**Keywords:** Sunn pest, supercooling point, cold tolerance, polyols, diapause

## Abstract

As a serious pest of wheat, the Sunn pest, *Eurygaster integriceps* Puton (Hem.: Scutelleridae), is prevalent in Iran. This pest belongs to univoltine species and tends to estivate and overwinter in high altitudes of nearby mountains as diapausing adults. The economic importance of the crop was attacked by this pest, i.e., wheat led the authors to study the physiological adaptations of these diapausing adults, that is, changes in the supercooling point (SCP), in the accumulation of cryoprotectants, and in the activities of the related enzymes in relation to diapause development. The mean SCP of the diapausing adults was found to be −8°C. The lowest SCP, i.e., approximately −11°C, was observed in the middle of diapause, October, when the highest cold hardiness was also interestingly recorded. This finding proposed that SCP depression could be a feasible cold-tolerance strategy for diapausing adults. The sugar content was high in the initiation and at the termination of diapause and was low during diapause maintenance. These sugar reserves were most likely utilized to be converted to glycogen and lipid during diapause maintenance as a survival strategy. The changes in the glycogen and lipid contents were inversely proportional to the changes in the total sugar content. The authors also found that the changes in the glycogen content were directly proportional to those in the low-molecular-weight carbohydrates (e.g., glycerol and trehalose) and in the diapause development. This finding underlined the role of the low-molecular-weight carbohydrates, such as the cryoprotectants, in enhancing the cold tolerance of the given insect. In this study, the diapause-associated changes in the activities of α-amylases and proteases were also investigated. The results showed that the enzyme activities were related to diapause development and cold-tolerance enhancement. The highest enzyme activity was observed in September. Since the overwintering adults of the Sunn pest could not tolerate temperatures below their SCPs, they were grouped in the freeze-intolerant species.

## Introduction

The ambient temperature can directly affect different stages of the life cycle of insects, as an ectothermic group of animals, including growth, development, reproduction, survival, and distribution (Régničre et al., 2012; Jaworski and Hilszczański, 2014). A large number of insects living in the places where the environment faces seasonal variations tend to enter diapause to circumvent adversities in the environment. The mechanism adopted by the insects to avoid adversities in the environment is known as diapause (specified arrest of development), during which temperature, photoperiod, and/or food quality encourage some pre-programmed physiological changes, thus leading to the suppression of metabolism, the enhancement of resistance to cellular stress, and the onset of dormancy in advance of unfavorable environmental conditions (Poelchau et al., 2013; Roncalli et al., 2018). The suppression of insect metabolism and the cessation of direct development lead to the regulation of the timing of growth, maturation, and reproduction. Finally, the optimum conditions in the environment can eventually harmonize the development of insects (Roncalli et al., 2018).

**FIGURE 1:**
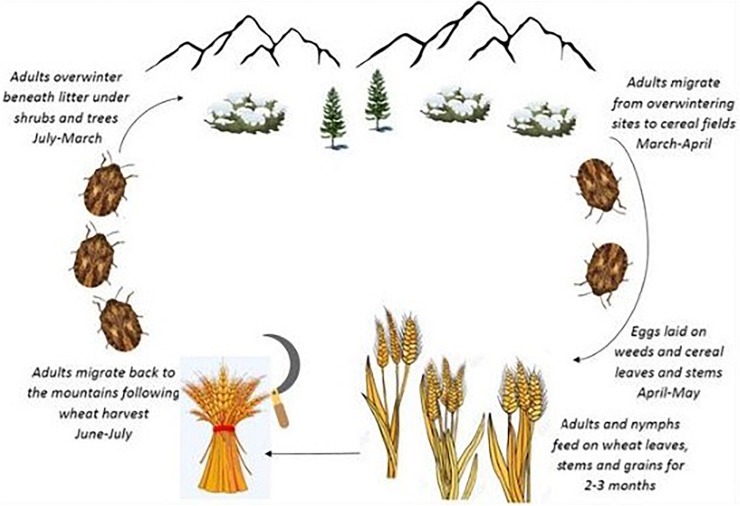
Life cycle of *Eurygaster integriceps* “adapted with permission” from Davari and Parker (2018).

The Sunn pest, *Eurygaster integriceps* Puton (Hem.: Scutelleridae), is a key pest of wheat in the west and central Asia and remains a serious pest in wheat-producing areas of Iran. *E*. *integriceps* is a univoltine pest overwintering as a diapausing adult. The diapausing adults migrate to mountains during June–July and following wheat harvest and overwinter beneath litter layers under shrubs and trees. Moreover, in this species, diapause is induced in advance of the advent of harsh environmental conditions. The overwintering adult migrates back to the cereal fields in the following spring (March–April) (Iranipour et al., 2010; Parker et al., 2011; Davari and Parker, 2018) (Figure 1).

One of the cryptobiotic states observed during the ontogenesis of insects is diapause, in which the metabolic processes markedly reduce. Within this period, the species undergo diapause as a cryptobiotic (deep metabolism suppression) phase of the insect’s ontogenesis, during which direct development is arrested, and the species shows no visible signs of life (Koštál, 2006; Hand et al., 2016; Diniz et al., 2017). The diapause periods in insects can be categorized as pre-diapause, diapause, and post-diapause, each of which has several subcategories (Koštál, 2006). The first period, namely, pre-diapause (e.g., May–June), includes two sub-phases, i.e., induction and preparation, during which the species prepares itself for the suspended development or the diapause. This period is followed by the diapause, which itself includes three sub-phases, i.e., initiation (July–September in our research), maintenance (October–November in our research), and termination (December–March in our research). Initiation is mainly characterized by the arrest of development and strong suppression of metabolism period. The morphogenetic, metabolic, and reproductive traits of the insects then halt in the next subcategory, i.e., maintenance, lasting for a couple of weeks or months. Termination, eventually, is marked by the renewal of the signs of life in the species, resulting from photoperiod, several endogenous processes, and temperature changes in the environment. Here, metabolism quickly normalizes (Koštál, 2006; Hand et al., 2016; Diniz et al., 2017).

Cold hardiness or cold tolerance is the ability of an insect to resist long- or short-term exposure to low temperatures (Lee et al., 1991; Sinclair et al., 2015). The extent to which ambient temperature affects the survival and reproduction of insects is determined by the development of cold hardiness in insects (Feng et al., 2018). Diapause and cold hardiness are both adaptation strategies exploited by most insects to survive from sub-zero temperatures in temperate zones (Denlinger, 1991; Storey and Storey, 2004). However, the relationship between these two strategies is not clear. In some insects, these two are independent phenomena (Goto et al., 2001; Khanmohamadi et al., 2016; Mollaei et al., 2016; Mohammadzadeh et al., 2017), while in some other insects, cold hardiness is a component of the diapause syndrome (Milonas, 1999; Bemani et al., 2012; Heydari and Izadi, 2014; Cira et al., 2018). In most insects, development of cold hardiness is highly associated with some physiological adaptations, e.g., synthesis and accumulation of low-molecular-weight carbohydrates and polyols (cryoprotection) (Behroozi et al., 2012; Vrba et al., 2017; Mohammadzadeh and Izadi, 2018a, b; Izadi et al., 2019), synthesis of antifreeze proteins or carbohydrates (Costanzo and Lee, 2013; Khanmohamadi et al., 2016; Mollaei et al., 2016), use of cryoprotective dehydration (Elnitsky et al., 2008; Clark et al., 2009; Worland et al., 2010), and regulation of ice nucleation (Costanzo and Lee, 2013).

The temperature at which fluids in the body of insects start freezing is defined as the supercooling point (SCP). The SCP is experimentally determined by the measurement of the exotherm released by the latent heat of water fusion during cooling exposure. Investigations on the strategies adopted by insects to develop cold tolerance usually begin with the preliminary measurement of SCP (Sinclair et al., 2015; Ditrich, 2018). These strategies can be categorized into three groups: chill-susceptible strategy (in which insects die even after a brief exposure to sub-zero temperatures), freeze-avoidance strategy (in which insects can tolerate moderate to high sub-zero temperatures but without internal ice formation, i.e., supercooled state), and freeze-tolerance strategy (in which insects can tolerate internal ice formation) (Sinclair, 1999; Sinclair et al., 2015).

Three main digestive enzymes, including proteases, amylases, and lipases, facilitate the digestion of macromolecules existing in the food. Amylase, as a glycoside hydrolase enzyme, functions as a catalyzer to hydrolyze the glycosidic bonds of polysaccharides, such as starch and glycogen, thus breaking them down into mono and/or disaccharides. Depending on the type of bond they form, namely, α or β, this group has two major enzymes, that is, α-amylase and β-amylase. Another hydrolase enzyme is proteases, catalyzing the hydrolysis of peptide linkages of proteins and releasing amino acids from protein. Both of the aforementioned enzymes are vital for digesting food efficiently (Nation, 2015).

Cryoprotectants can be synthesized and accumulated to increase cold hardiness of many insects, the most prevalent of which include polyhydric alcohols (e.g., glycerol), low-molecular-weight carbohydrates (e.g., trehalose), and amino acids (Fuller, 2004). Amino acids can lower the non-colligative freezing points of water, thus functioning as an antifreeze compound. It may also increase the solute concentration of fluids of the body of insects, thereby acting as a cryoprotectant (Zachariassen, 1985; Li, 2012). Antifreeze (IFP) or ice-binding proteins were categorized into three major groups (Duman, 2015), that is, ice-binding proteins, which typically do their antifreeze actions by great thermal hysteresis, antifreeze proteins, which generally follow their antifreeze actions by low thermal hysteresis, and ice-nucleating proteins, which seemingly prevent intracellular ice formation via forming a template around the ice. Thus, the formation of ice securely occurs in the extracellular fluid of the body, where no fatal intracellular ice crystallizes. As the simple biomolecules, cryoprotectants, however, are generally created through the metabolic activities of a number of enzymes (Storey and Storey, 1991; Walters et al., 2009). The main classes of cryoprotectants, polyols, are usually formed in tissues of insects, when glycogen, as the substrate, is employed in various enzymatic biochemical processes (Lee, 2010). Different media, including pyruvate, oxaloacetate, α-ketoglutarate, and 3-phosphoglycerate, can form amino acids via different enzymatic biochemical mechanisms (e.g., citric acid cycle). In this study, the authors made an effort to study the impact of two enzymes, i.e., amylase and protease, to understand how these enzyme activities might relate to cold tolerance in the overwintering adults of *E. integriceps*. The correlation between diapause development and cold hardiness was also examined. Additionally, the influence of some metabolites upon cold hardiness of *E. integriceps* was studied within the 9-month period of diapause.

## Materials and Methods

**FIGURE 2:**
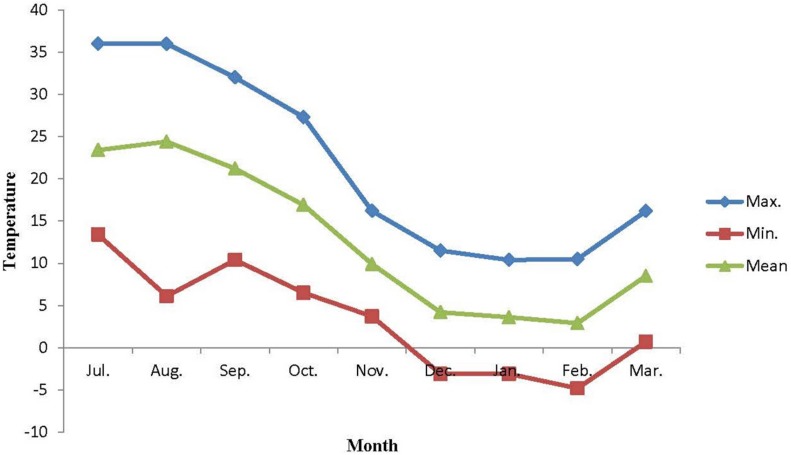
Ambient temperature of overwintering habitats of *Eurygaster integriceps* during different overwintering months of 2015–2016.

The mature pests of *E. integriceps* were gathered monthly (from July to March) from overwintering sites in Lorestan province (48°,19′,22″ N, 23°,50′,11″ E, and altitude of 1,943 m), Iran. The conditions under which the pests were preserved prior to sampling were constant. Data as to temperature changes (Figure 2) in the environment were provided by the Data Processing Center of Iran Meteorological Organization (IMO), situated close to the sampling site.

### Determination of SCP

The SCPs of adults (*n* =6–10 individuals per month) were determined using a thermocouple (NiCrNi probe) connected to an automatic temperature recorder, Testo 177-T4 (Testo, Germany). The temperature was recorded every 30 s, and the data were then read using Comsoft 3 Software. Each specimen was attached to the thermocouple by means of an adhesive tape and was placed inside a programmable refrigerated chamber (Gotech; GT-7005-A; Taiwan), whose temperature was lowered at a rate of 0.5°C per minute. The SCP was taken as an abrupt temperature increase occurring with the release of the latent heat of crystallization (Mohammadzadeh and Izadi, 2016).

### Cold Tolerance Assay

Cold tolerance of the adults was assayed by cooling individual adults (*n* = 17–58) to 5, 0, −5, or −10°C in Petri dishes. The Petri dishes were placed in a programmable refrigerated test chamber (Gotech; GT-7005-A; Taiwan). The temperature was lowered from 25°C to the desired treatment temperature at a rate of 0.5°C per minute. At each set temperature, the adults were removed after 24 h and returned to an optimal temperature (25 ± 1°C). The live and dead adults were counted after 24 h (Mohammadzadeh and Izadi, 2016).

### Weight and Water Quantification

The adults (*n* = 6 per month) were separately weighed and dried in an oven at 65°C for 72 h. The water content (w/w) was obtained from subtracting dry weight from fresh weight and then dividing the result by fresh weight (Lehmann et al., 2012; Heydari and Izadi, 2014).

### Total Body Sugars Quantification

The total simple sugars (monosaccharides and disaccharides) were determined using the anthrone reagent method proposed by Warburg and Yuval (1997). Briefly, individual adults were weighed and homogenized with a homogenizer (Teflon pestle; 0.1 mm clearance) in 200 μl of 2% Na_2_SO_4_. In order to extract the simple sugars, 1,300 μl of a chloroform–methanol mixture (1:2) was initially added to the homogenate and centrifuged at 7,150 × *g* for 10 min. Next, 300 μl of the supernatant was mixed with 200 μl of distilled water and reacted with 1 ml of the anthrone reagent (500 mg of anthrone dissolved in 500 ml of concentrated H_2_SO_4_) for 10 min at 90°C. The amount of total simple sugars was determined at 630 nm using a spectrophotometer (T60U; Harlow Scientific, United States). Glucose (Sigma) was used as a standard. This experiment was carried out per month using six adults, each as a replicate (Heydari and Izadi, 2014; Mohammadzadeh and Izadi, 2016).

### Glycogen Determination

The pellet obtained from the analysis of total body sugars was used for the determination of glycogen content. To remove the possible remnants of sugar, the pellet was washed with 400 μl of 80% methanol and mixed with 250 μl of distilled water. This mixture was heated at 70°C for 5 min. Next, 200 μl of the solution was removed and reacted with 1 ml of the anthrone reagent (600 mg of anthrone dissolved in 300 ml of the concentrated H_2_SO_4_) for 10 min at 90°C. The optical density was read at 630 nm on a spectrophotometer (T60U; Harlow Scientific, United States). Glycogen (Sigma) was used as a standard. This experiment was carried out per month using six individuals, each as a replicate (Heydari and Izadi, 2014; Mohammadzadeh and Izadi, 2016).

### Low-Molecular-Weight Carbohydrates and Polyol Assay

To determine low-molecular-weight carbohydrates and polyols (trehalose, glucose, glycerol, and myo-inositol), the adult bugs were weighed, homogenized in 1.5–2 ml of 80% ethanol with the pre-cooled homogenizer (Teflon pestle), having a clearance of 0.1 mm, and centrifuged at 12,000 × *g* for 15 min. The supernatant was evaporated in a vacuum drying oven at 40°C and then resuspended in 1 ml of HPLC-grade water. The samples were passed through a 20-μm syringe filter and analyzed by high-performance liquid chromatography (Knauer, Berlin, Germany) equipped with a carbohydrate column having 4-μm particle size (250 mm × 4.6 mm, I.D.; Waters, Ireland). Acetonitrile–water (70:30) was used as eluent. The elution speed was 1 ml/min^–1^, and the separation was achieved at 40 ± 1°C. Twenty microliters of the whole-body extracts along with the standard of each carbohydrate from 1,500 to 5,500 ppm were run. This experiment was carried out per month using six individuals, each as a replicate (Heydari and Izadi, 2014; Mohammadzadeh and Izadi, 2016).

### Enzymes Assay

#### Preparation of the Samples

The adult bugs were anesthetized on ice slurry and decapitated. The midgut was gently removed with the aid of a stereomicroscope (Stemi SV6 ZEISS; Germany) and placed in 1.5-ml microtubes containing 1.0 ml of cooled distilled water. Out of each sample, 10 midguts underwent the mentioned process. The samples were homogenized and centrifuged at 15,000 × *g* for 15 min at 4°C. The supernatant was used for the enzymatic assays (Borzoui et al., 2015; Mohammadzadeh and Izadi, 2016).

#### Amylase Activity Assay

Amylolytic activity of α-amylase was determined based on the procedure proposed by Bernfeld (1955). A mixture of 40 μl of 1% freshly prepared starch solution, 500 μl of 20 mM phosphate buffer (pH 7), and 20 μl of enzyme extracts was prepared and incubated for 30 min at 37°C. Then, 100 μl of the 3,5-dinitrosalicylic acid (DNS) reagent was added to the mixture and heated in boiling water for 10 min to stop the reaction. The optical density (OD) was measured at 540 nm using a spectrophotometer (T60U; Harlow Scientific, United States). Maltose (Sigma) was used as a standard, and the amylolytic activity was determined from a standard curve. The experiments were run in five replicates with blanks containing no enzyme extracts.

#### Protease Activity Assay

The azocasein method was used to assay the digestive proteolytic activity of *E. integriceps* adults collected during a period of several months (Gatehouse et al., 1999; Elpidina et al., 2001), albeit a number of modifications were made based on Mohammadzadeh and Izadi (2018b). A mixture of 10 μl of midgut homogenate, 40 μl of glycine–NaOH buffer (pH 10), and 50 μl of 2% azocasein substrate was incubated for 60 min at 37°C. By adding 100 μl of 30% trichloroacetic acid, the reaction halted. The mixture was held for 30 min at 4°C and then centrifuged at 10,000 × *g* for 15 min. The supernatant was dissolved in an equal volume of 1.0 M NaOH. The absorbance was recorded at 405 nm. All assays were run in five replicates with blanks containing trichloroacetic acid (TCA).

### Statistical Analysis

The Kolmogorov–Smirnov test was initially performed to examine the normality of the studied data. The Levene’s test was then used to indicate homoscedasticity. The one-way analysis of variance (ANOVA) and the *post hoc* Tukey’s test (P = 0.05) were subsequently run to compare multiple treatments. For non-normally distributed SCP data, the Mann–Whitney U and Kruskal–Wallis tests were additionally administered to trace differences observed in data.

## Results and Discussion

### The SCP Changes During Diapause

**FIGURE 3:**
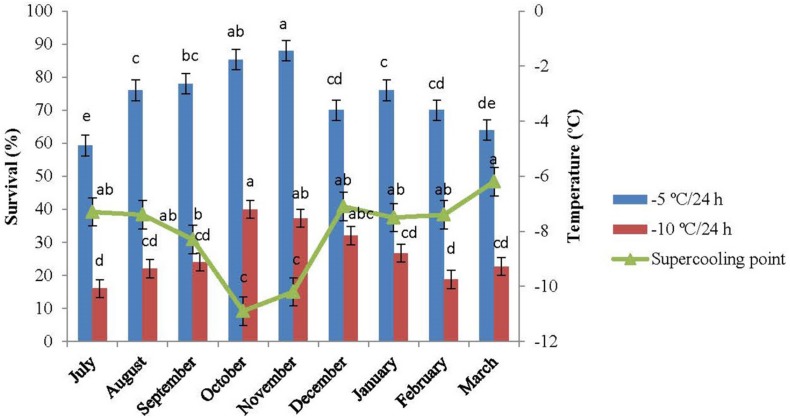
Changes in the supercooling point and survival of *Eurygaster integriceps* during different months of diapause. Means ± SE: for each month, means followed by the same letter are not significantly different (*P* > 0.05) (each experiment was carried out with six individuals each month).

The SCP of the overwintering adults were measured monthly from July 2015 to March 2016. The changes in SCP are presented in Figure 3. The SCPs of the diapausing adults of *E. integriceps* ranged from −7.3°C (traced in July) to −6.2°C (detected in March), exhibiting a U-shaped curve. The SCP decreased from approximately −7°C (in July and August) to about −8°C (in September) and finally reached the lowest level in the diapause maintenance, i.e., in October and November (-1.9°C and −10.2°C, respectively). From December onward, SCP increased and reached the highest level (about −6°C) at the approach of spring. The cold hardiness of *E. integriceps* was also investigated in the altitude of Ateshgah Karaj-Iran (see Baghdadi et al., 2001), the results of which demonstrated that SCPs varied from −12.9°C (in early winter of 1998–1999) to −6.7°C (in 1999–2000). Similarly, the cold-tolerance strategies of the aforementioned pest were studied in altitude of Ghara-aghaj Varamin-Iran (Baghdadi, 2007), and SCPs of the overwintering species under investigation were measured. In this research, SCP reported for the coldest month of the year was −5°C. On the other hand, Cira et al. (2018) studied changes in SCPs of the diapausing adults of *H. halys* and concluded that SCP in October was significantly higher than that in other months of the diapause. Košál and Šimek (2000) investigated the overwintering strategy of *Pyrrhocoris apterus* (Heteroptera: Pyrrhocoridae) and reported the highest level of SCP at the termination of the diapause of the adult bugs. Overall, the range of SCP changes of *E. integriceps* was relatively low during diapause, and the mean SCP was the lowest in the phase of the diapause maintenance of the adults. This interpretation was compatible with that reported by Hodkova and Hodek (1997) indicating that SCP of the overwintering adults of *P. apterus* was about −7°C at the onset of the pre-diapause and decreased to about −12°C in the overwintering adults (January and February). Bastola and Davis (2018) also showed that the mean SCP of the overwintering adults of the redbanded stink bug, *Piezodorus guildinii* (Westwood) (Hem.: Pentatomidae) changed from −8.3°C (in March) to −11.0 ± 0.2°C (in January). Moreover, Cira et al. (2016) reported limited changes in SCP during summer, fall, and winter among different geographical populations of *Halyomorpha halys* (Hem.: Pentatomidae). Elsey (1993) reported that SCP of the diapausing and non-diapausing adults of *Nezara viridula* (L.) (Hem.: Pentatomidae) ranged from −10.4°C to −11.7°C. This limited range of SCP suggested that the diapausing adults of *E. integriceps* might employ more than one survival strategy. During the overwintering stages, the cold tolerance of those insects, which are physiologically incapable of synthesizing and accumulating cryoprotectants, is usually accompanied by the SCP depression. That is to say, the SCP expansion is the main cold-tolerance strategy deployed by this group of insects (Mollaei et al., 2016). However, in the insects with the capability of cryoprotectant biosynthesis and accumulation, the SCP changes are commonly limited, and the development of cold tolerance is commonly associated with cryoprotectant accumulation and/or SCP degradation (Zachariassen, 1985; Heydari and Izadi, 2014; Sinclair et al., 2015; Ditrich et al., 2018).

The results of this study showed that the association between minimum ambient temperature and the mean SCP was not strong and straightforward. Likewise, the values for SCP of the diapausing adults of *E. integriceps* did not follow a direct seasonal trend. The lowest SCP was achieved in October, whereas the lowest temperature was recorded in February. The same results were released by Ditrich et al. (2018). They stated that SCP of the linden bug, *P. apterus* could strongly correlate with the ambient temperature; however, there was no direct relationship between the lowest ambient temperature and SCP. Mollaei et al. (2016) also mentioned that the SCP values and the ambient temperatures did not follow the same direct seasonal trend in the overwintering larvae of *Kermania pistaciella* (Lep.: Tineidae). On the other hand, Ditrich and Koštál (2011) proposed a strong correlation between SCP and lower lethal temperatures among nine species of the semi-aquatic bugs (Hem.: Gerromorpha). In the current study, SCP was approximately 5°C lower than the minimum ambient temperature. In the research done by Ditrich et al. (2018), the SCP of *P. apterus* was about 10°C lower than the minimum ambient temperature.

### Cold-Tolerance Strategies

When the pre-diapausing and diapausing adults of *E. integriceps* were exposed to −5°C and −10°C/24 h, survival increased with a decrease in SCP and reached the highest level in October and November with the lowest SCP. From November onward, survival decreased with an increase in SCP, the rate of which in March corresponded to that in July (Figure 3). Cold tolerance in the phase of diapause maintenance was significantly higher than that in the initiation and at the termination of the diapause. The least survival rate was observed in the initiation and at the termination phases of the diapausing adults with survival rates of 16.0 and 18.7%, respectively, following the exposure to −10°C/24 h. Almost no adults survived after 24-h exposure to −15°C.

The increase in cold tolerance of the diapausing adults was highlighted by a remarkable decrease in the range of SCP. This finding indicated that SCP had a predictive value and could be recommended as a suitable means for the determination of the survival of the overwintering adults. This was in agreement with the results reported by Kalushkov and Nedvěd (2000) and Ditrich et al. (2018), suggesting the use of SCP as an appropriate index of cold hardiness in the overwintering adults of *P. apterus*.

One of the tactics the overwintering adults of *E. integriceps* exploited to survive in cold conditions was migration to higher altitudes, thus entering diapause in the aggregate populations under shelter shrubs. Besides, the results of the current work suggested seasonal cold acclimation as a further means by which the diapausing adults of *E. integriceps* reduced the lethal effects of exposure to low temperatures. This is typical of many temperate insects (Cira et al., 2016). Since the overwintering adults of *E. integriceps* could not survive temperatures below their SCPs, this pest was considered to be a freeze-intolerant species. The Linden Bug, *P. apterus*, was also reported to be a freeze-intolerant species (Kalushkov and Nedvěd, 2000; Cira et al., 2016).

### Seasonal Changes in Water Content and Body Mass of Adults

**FIGURE 4:**
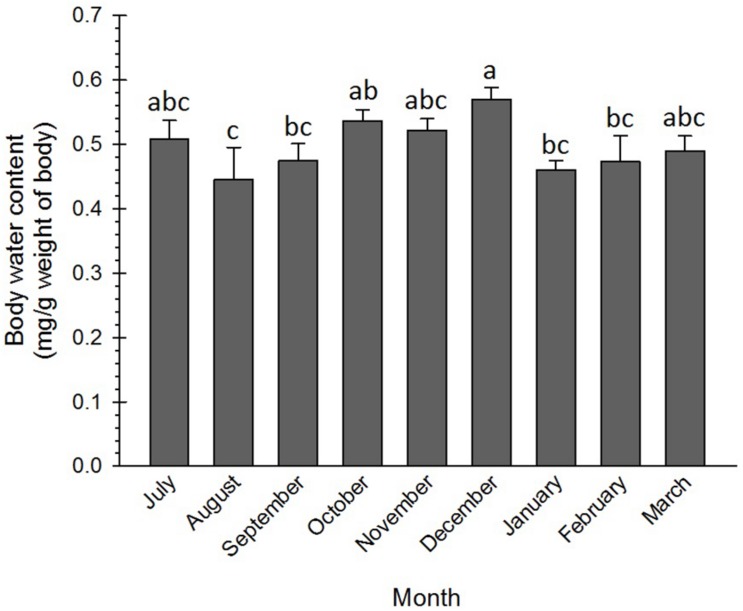
Changes in water content of *Eurygaster integriceps* during different months of diapause. Means ± SE: for each month means within a column followed by the same letter are not significantly different (*P* > 0.05) (each experiment was carried out with 6 individuals each month).

The water content of the adult insects slightly varied from the beginning to the end of the diapause. High water content was recorded in three phases, i.e., initiation (July), maintenance (October–December), and termination (March). The difference in the water content of these phases of the diapause was not significant. However, the water content in December was significantly more than that in August–September and in January–February (Figure 4). In most of the insects, diapause termination is associated with an increase in water content to resume morphogenesis (Frankos and Platt, 1976; Hodek, 2003). In the current research, the water content of the diapausing adults of the Sunn pest at the diapause termination (March) increased and corresponded to that in the diapause initiation (July). In coincidence with this result, Xiao et al. (2015) found that water content in the non-diapausing pupae of *Pieris melete* (Lep.: Pieridae) was significantly more than that in the diapausing pupae. Besides, the changes in the water content of the diapausing pupae were almost negligible during the diapause initiation and maintenance phases but substantially increased at the diapause termination phase. Heydari and Izadi (2014) also found an appreciable difference between the water content of the diapausing (62%) and the non-diapausing (80%) larvae of the carob moth, *Ectomyelois ceratoniae* Zeller (Lep.: Pyralidae).

**FIGURE 5:**
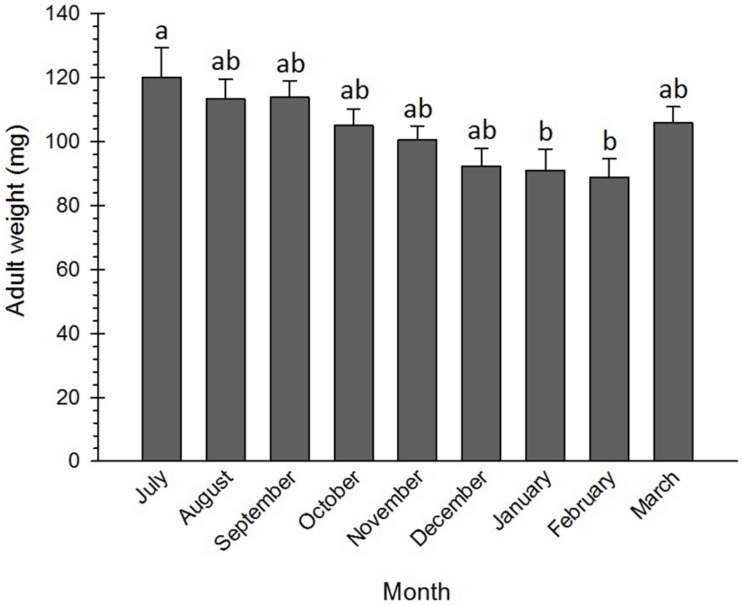
Changes in body mass of *Eurygaster integriceps* during different months of diapause. Means ± SE: for each month, means within a column followed by the same letter are not significantly different (*P* > 0.05) (each experiment was carried out with six individuals each month).

Numerous physiological and cellular survival mechanisms were exploited by insects when exposing to low temperatures, among which freeze tolerance, freeze avoidance, and cryoprotective dehydration were the most important ones (Clark et al., 2009; Sinclair et al., 2015). Cryoprotective dehydration is one of the least frequently used strategies insects deploy to adapt to cold conditions. By exploiting this tactic, insects become almost anhydrobiotic due to a decrease in their water content. This adaptation mechanism may be used by both freeze-avoiding and freeze-tolerating species, including *Graphosoma lineatum* (Šlachta et al., 2002), *Belgica antarctica* (Elnitsky et al., 2008), *Megaphorura arctica* (Worland et al., 2010), *Cucujus clavipes puniceus* (Sformo et al., 2010), *Hypogastrura viatica*, *Folsomia quadrioculata*, *Oligaphorura groenlandica* and *M. arctica* (Sørensen and Holmstrup, 2011), *Dendrolimus tabulaeformis* (Shao et al., 2018), and *P. melete* (Xiao et al., 2015), to survive cold stress via increasing osmolyte concentration. No significant differences were detected between the weights of the adults from July to December, although their weights in January and February were significantly lower than those in July (Figure 5). The pre-diapause insects generally accumulate reserves and utilize these energy sources during the metabolically depressed phase of the diapause (Hahn and Denlinger, 2011). Therefore, lowering the weight at the diapause termination phase might be attributed to the depletion of energy resources and the decrease in water content. Brent et al. (2013) found that the weight of the non-diapausing plant bug, *Lygus hesperus* Knight (Hem.: Miridae), was discernibly larger than that of the diapausing one.

### Biochemical Analysis

#### Total Body Sugar and Glycogen and Lipid Contents

In the initiation phase of the diapause, i.e., July, the total body sugars, forming 12.14 mg/g of the weight of the body, were at the highest level. These amounts decreased toward December and reached the lowest level in the diapause maintenance. From December onward, the total body sugars increased (Table 1). That is to say, the sugar reserves were most likely utilized during the diapause initiation and termination when the ambient temperature was almost suitable. The adult insects, however, migrated to the diapausing habitats and stayed there in a dormant state. The changes in the glycogen and lipid contents were inversely proportional to those in the total sugar content (Table 1). In the diapause initiation, glycogen and lipid contents (8.68 and 4.38 mg/g of the weight of the body, respectively) were at the lowest level and increased with the diapause development; from November onward, the contents reached the highest level. These results showed that the overwintering adults of *E. integriceps* altered their metabolic energy reserves during different phases of the diapause. In accordance with this result, Amiri and Bandani (2013) demonstrated that the energy reserves of the Sunn pest varied from the diapause initiation to the diapause maintenance. Based on the findings of the current study, the overwintering adults of *E. integriceps* mostly relied on sugars as the main energy reserve in the diapause initiation and switched to another fuel source (lipids) in the diapause maintenance when metabolism was fully repressed. Amiri and Bandani (2013) also reported that the diapausing adults of the Sunn pest accumulated lipids for their metabolic needs during the diapause maintenance and termination. Most of the insects generally suppress their metabolism during overwintering. Therefore, before the diapause initiation, to survive in the suppressed metabolic conditions, insects must equip themselves physiologically. Moreover, to provide the energetic demands of the post-diapause development and the reproduction or to perform metabolically expensive functions, such as migration, overwintering insects must manage their energy resources during diapause maintenance (Hahn and Denlinger, 2011; Tan et al., 2017; Sinclair and Marshall, 2018). Lipids and glycogen are the two main sources satisfying the energy demands of the overwintering insect (Arrese and Soulages, 2010; Sinclair and Marshall, 2018). An increase in glycogen content and a decrease in total body sugar content indicate an interconversion of these two carbohydrates and diapause development. Also, the increased glycogen content can provide the necessary cryoprotectants and energy reserves. Overall, the deceased sugar content might denote that the adults were consuming sugar as the diapause went on, and the increased glycogen and lipid contents could indicate that these two forms of nutrients were stored in the diapausing adults. Moreover, the increased glycogen and lipid contents might be derived from the consumed sugar. In the diapausing larvae of *Dendrolimus tabulaeformis* (Lep.: Lasiocampidae), the glycogen content reached its peak in November and then decreased (Shao et al., 2018). Lu et al. (2014) suggested that increased metabolites might be employed by the diapause-destined pupae of the cotton bollworm, *Helicoverpa armigera* (Hübner) (Lep.: Noctuidae), as an energy source, metabolic intermediates, and cryoprotectants. In the current study, glycogen content increased during the diapause termination, while the changes in the total lipid content were not significant (Table 1). However, in most insects, the energy levels of the metabolic activities rapidly increase with diapause termination to meet the energy demands for the post-diapause growth and reproduction (Lu et al., 2014; Sinclair and Marshall, 2018). Batz and Armbruster (2018) observed that the embryonic diapause of the Asian tiger mosquito, *Aedes albopictus* (Skuse) (Dip.: Culicidae), was associated with increased energy storage (lipids) and with lipogenesis. Shao et al. (2018) documented that the lipid content of the diapausing larvae of *D. tabulaeformis* gradually decreased during the diapause initiation, remained stable in the diapause maintenance, and reached amounts comparable to those observed in September at the diapause termination. Zhang et al. (2019) showed that the overwintering females of *Culex pipiens pallens* (L.) (Dip: Culicidae) accumulated more lipids during the diapause initiation and maintenance than during the non-diapausing phase. Employing lipids as a source of energy during diapause was also found in some other insects (Han et al., 2008; Behroozi et al., 2012; Bemani et al., 2012; Heydari and Izadi, 2014).

**TABLE 1:** Chemical content of overwintering adults of *Eurygaster integriceps* (*n* = 6) in 2015–2016.

Month	Chemical contents (mg/g body weight)			
	Total sugars	Glycogen	Lipid	Protein
July	12.14 ± 0.48^a^	8.68 ± 0.14^c^	4.38 ± 0.23^cd^	2.16 ± 0.04^bc^
August	6.23 ± 0.22^c^	8.63 ± 0.31^c^	4.24 ± 0.13^d^	2.09 ± 0.10^bc^
September	3.09 ± 0.08^e^	9.08 ± 0.22^bc^	4.43 ± 0.04^cd^	1.86 ± 0.05^c^
October	3.69 ± 0.07^de^	10.84 ± 0.18^a^	4.77 ± 0.03^c^	1.73 ± 0.07^c^
November	3.67 ± 0.12^de^	10.68 ± 0.25^ab^	5.28 ± 0.02^ab^	2.35 ± 0.09^ab^
December	3.97 ± 0.29^de^	11.61 ± 0.75^a^	5.42 ± 0.05^a^	2.51 ± 0.15^ab^
January	4.29 ± 0.07^d^	11.05 ± 0.27^a^	5.63 ± 0.12^a^	2.60 ± 0.13^a^
February	5.81 ± 0.01^c^	11.19 ± 0.38^a^	5.78 ± 0.08^a^	2.70 ± 0.05^a^
March	8.60 ± 0.07^b^	10.73 ± 0.28^a^	4.83 ± 0.11^bc^	2.73 ± 0.08^a^

*Means ± SE: means within a column followed by the same letter are not significantly different (P > 0.05).*

### Low-Molecular-Weight Carbohydrates (LMWC)

In this research, five potential cryoprotectants, i.e., trehalose, glycerol, sorbitol, myo-inositol, and glucose, were detected in the overwintering adults of *E. integriceps* from July to March. Out of these five LMWCs, glucose, glycerol, and trehalose were found to be the most prominent cryoprotectants in the overwintering adults. The amounts of these carbohydrates were at the lowest levels in the diapause initiation, reached the highest levels in the diapause maintenance, and decreased at the diapause termination (Table 2). Insects commonly synthesize and accumulate cryoprotectants during overwintering. An increase in the concentration of these metabolites results in elevated hemolymph viscosity (Toxopeus and Sinclair, 2018; Sinclair and Marshall, 2018; Toxopeus et al., 2019). Likewise, Shao et al. (2018) studied the changes in the concentration of trehalose, glucose, and glycerolin in the overwintering larvae of *D. tabulaeformis*. They concluded that trehalose was the main cryoprotectant, whose level reached the highest in November. However, glycerol content remained unchanged during the diapause maintenance, though it substantially rose in May. Glucose content was at the maximum level in January and then gradually decreased. The overwintering nymphs of the wolf spider *Pardosa astrigera* (Araneae: Lycosidae) accumulated a high level of glycerol and a small amount of myo-inositol (Tanaka and Ito, 2015). In the overwintering nymphs and the adults of the bush tick, *Haemaphysalis longicornis* (Acari: Ixodidae), the glycerol content showed a marked elevation (Yu et al., 2014).

**TABLE 2:** Carbohydrate contents of overwintering adults of *Eurygaster integriceps* in 2015–2016.

Time	Trehalose	Glycerol	Sorbitol	Myo-inositol	Glucose
July	0.24 ± 0.01^c^	0.63 ± 0.00^f^	0.01 ± 0.00^d^	0.09 ± 0.00^c^	0.50 ± 0.01^d^
August	0.24 ± 0.00^c^	0.63 ± 0.01^f^	0.02 ± 0.00^cd^	0.11 ± 0.00^bc^	0.38 ± 0.01^d^
September	0.26 ± 0.00^c^	0.75 ± 0.00^e^	0.02 ± 0.00^cd^	0.11 ± 0.00^bc^	0.43 ± 0.01^d^
October	0.42 ± 0.00^b^	0.89 ± 0.01^d^	0.04 ± 0.00^ab^	0.13 ± 0.01^b^	0.73 ± 0.02^c^
November	0.46 ± 0.00^a^	0.92 ± 0.00^cd^	0.05 ± 0.00^a^	0.18 ± 0.00^a^	0.88 ± 0.03^b^
December	0.47 ± 0.00^a^	0.94 ± 0.00^bc^	0.05 ± 0.00^a^	0.17 ± 0.00^a^	1.26 ± 0.01^a^
January	0.48 ± 0.01^a^	1.24 ± 0.01^a^	0.05 ± 0.00^a^	0.18 ± 0.00^a^	1.22 ± 0.01^a^
February	0.41 ± 0.01^b^	1.26 ± 0.01^a^	0.04 ± 0.00^ab^	0.16 ± 0.00^a^	0.92 ± 0.01^b^
March	0.25 ± 0.01^c^	0.97 ± 0.00^b^	0.03 ± 0.00^bc^	0.12 ± 0.00^b^	0.74 ± 0.02^c^

*Means ± SE: means within a column followed by the same letter are not significantly different (P > 0.05).*

Interestingly, the SCP reducing trend and the cold-hardiness development patterns, observed during the course of the diapause maintenance of *E. integriceps*, were consistent with the tendency found in these insects for increasing their cryoprotectant levels. Most of the diapausing insects enhanced their SCP and cold tolerance via regulating physiological–biochemical processes, thus resulting in cryoprotectant synthesis and accumulation. In the current research, the least SCP and the maximum cold hardiness in the diapause maintenance corresponded to the highest amounts of glucose, glycerol, and trehalose. That is, the diapausing adults employed these metabolites as cryoprotectants to enhance their cold tolerance. The diapausing larvae of *D. tabulaeformis* regulated their SCP capacity and cold tolerance by the accumulation of trehalose and glucose (Shao et al., 2018). Insects living in the areas having severe ambient temperature during winter (temperate region) resort to either freeze-tolerance or freeze-intolerance mechanisms to survive in cold conditions (Storey and Storey, 2004; Lee, 2010; Sinclair et al., 2015). Similar to many other freeze-intolerant species, the diapausing adults of *E. integriceps* increased their SCP and cold hardiness via the accumulation of antifreeze cryoprotectants, such as glycerol, trehalose, and glucose, having colligative effects on their internal body fluids. Ishiguro et al. (2007) documented that the enhancement of cold hardiness in the overwintering larvae of rice stem borer, *Chilo suppressalis* (Walker) (Lep.: Crambidae) was associated with the elevation of glycerol level. Kalushkov and Nedvěd (2000) found a correlation between SCP and the cold hardiness of the diapausing adults of *P. apterus*. However, the results of the study done by Rozsypal et al. (2018) did not support the current findings. They found that increased metabolite concentration had no substantial effect on the cold hardiness of the bean bug, *Riptortus pedestris* (Fabricius) (Hem.: Alydidae).

The authors of the current study observed a direct correlation between the changes in glycogen content and those in LMWCs and diapause development. This finding was contrary to what they previously observed in another study, where the glycogen and LMWC contents did not follow the same increasing/decreasing trend, and glycogen was subsequently regarded as a source of cryoprotectants (Bemani et al., 2012; Heydari and Izadi, 2014; Khanmohamadi et al., 2016). The results of the present study indicated that there was a significant correlation among developing diapause, accumulating cryoprotectants, and increasing cold tolerance. That is to say, the diapause maintenance in the overwintering adults of *E. integriceps* was tightly associated with the enhancement of cold tolerance.

### Changes in the Enzyme Activity

**FIGURE 6:**
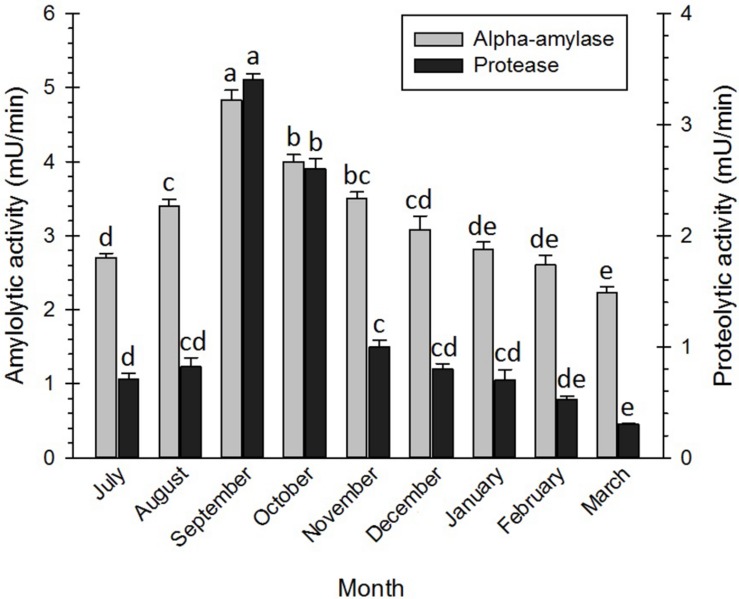
Changes in enzyme activity of *Eurygaster integriceps* during different months of diapause. Means ± SE: for each month, means within a column followed by the same letter are not significantly different (*P* > 0.05) (each experiment was carried out six times each month with 10 guts for each replication).

In this study, the activities of two enzymes, i.e., α-amylase and protease, were investigated during the diapause of the adult bugs. The results (Figure 6) showed that diapauses development was closely associated with enzyme activities. The activities of α-amylases in the initiation and at the termination of the diapause were generally higher than those of proteases in the mentioned phases. However, the activities of the enzymes increased with the diapause development and reached the highest levels in September. From September onward, the activities of the enzymes decreased and reached the lowest levels at the diapause termination (February–March). Similarly, Dmochowska et al. (2013) documented that the amylase activities significantly reduced at the end of the diapause of the red mason bee, *Osmia rufa* (Linnaeus) (Hym.: Megachilidae). Abraham et al. (1992) also hypothesized that the amylase activities in the diapausing strain of the silkworm, *Bombyx mori* L, were negligible, whereas the given activities substantially increased in the non-diapausing strain of the silkworm. Likewise, Shappirio (1974) reported higher activities in all studied enzyme systems of the diapausing pupae of *B. mori*. In *E. plotnikovi* Nikol’skaya (Hym.: Eurytomidae), however, the activities of α-amylase markedly differed in the diapausing and the non-diapausing larvae (Mohammadzadeh et al., 2017). In another study, it was demonstrated that the genes encoding the protease of *Leptinotarsa decemlineata* (Say) (Col.: Chrysomelidae) were down-regulated in the diapausing adults and relatively up-regulated in the non-diapausing adults (Yocum et al., 2009). In September, the adult bugs migrated from the temporal shelters to the overwintering habitats in the nearby mountains and hills. This behavior coincided with a decrease in total sugar content, an increase in LMWCs, glycogen, lipids, and proteins, and enhancement of the enzyme activities. By studying the obtained figures of the research, the authors detected that protease enzymes were far more active than amylase enzymes, suggesting that amino acids (e.g., proline, arginine, and serine) were significantly correlated with the cold tolerance of the diapausing adults, compared with sugars. Free amino acids could also contribute to an increase in cold hardiness by growing osmolality. They could also function as stabilizers for protecting cell membranes from degradation upon exposure to cold conditions (Goto et al., 2001; Bale et al., 2002; Feng et al., 2016; Koštál et al., 2016).

Glycosyl hydrolase enzymes, or α-amylases, are the main group of digestive enzymes that break down α-1,4-glycosidic linkages of a polysaccharide (e.g., starch and glycogen), resulting in, for example, maltose as one of the end products. As a substrate of α-glucosidases, maltose is, in turn, hydrolyzed into glucose (Da Lage, 2018). The reduction in total body sugars in the diapause initiation of *E. integriceps* might be attributed to its conversion into LMWCs and glycogen under the up-regulation of the catalytic activities of amylase enzymes. The hydrolysis of the peptide bonds in the polypeptide chain of proteins is catalyzed by a class of enzymes, i.e., protease or peptidase. These enzymes often perform in a cascade pathway (Kanost and Clem, 2012). In the current study, the activities of the protease enzymes reached the highest level in September, gradually decreased, and finally reached the lowest level at the diapause termination. Down-regulation of the digestive enzymes during the diapause maintenance could be attributed to the metabolic suppression during this phase of the diapause.

## Conclusion

The results of this study suggest that the diapausing adults of *E. integriceps* are freeze-intolerant species. This pest survived in very cold conditions of the diapause maintenance phase by accumulating LMWC cryoprotectants and by reducing SCP. The results of the current study can be employed to predict a model for the immigration of overwintering adults into the fields.

## Data Availability Statement

The datasets generated for this study are available on reasonable request to the corresponding author.

## Author Contributions

HH and HI conceived and designed the research and conducted the experiments. HI and MM contributed to the analytical tools and to the analysis of the data. HI wrote the manuscript.

## Conflict of Interest

The authors declare that the research was conducted in the absence of any commercial or financial relationships that could be construed as a potential conflict of interest.
